# A modified multi-patch technique for double-layered repair of ischemic posterior ventricular septal rupture

**DOI:** 10.1186/s40792-018-0426-3

**Published:** 2018-03-27

**Authors:** Takahiro Katsumata, Masahiro Daimon, Hayato Konishi, Shinji Fukuhara

**Affiliations:** Department of Thoracic and Cardiovascular Surgery, Osaka Medical College, 2-7 Daigaku-machi, Takatsuki, Osaka 569-8686 Japan

**Keywords:** Ventricular septal rupture, Myocardial infarction, Repair

## Abstract

**Background:**

The rupture of the posterior ventricular septum after acute inferior myocardial infarction is more challenging to repair than ruptures in other sites since it is less accessible and anatomically restricted. We described a modification of Daggett’s original technique of multi-patch repair of ruptured posterior septum.

**Case presentation:**

The technique was employed in the operation of a 67-year-old male who presented with severe heart failure at the 10th day after he developed inferior myocardial infarction. His ventricular septum had ruptured at the level between the posteromedial papillary muscle and the mitral annulus.

A large endoventricular patch covered separately over the locally patched septal defect and the ventriculotomy defect which was going to be roofed eventually with an external patch. Both defects were then individually closed in double layers, holding a single continuous patch in common. The common use of a single patch expedited multilayered closure of the left ventricular defects and could minimize geometric remodeling of the covered area. The patches on both the endocardial and the epicardial sides avoided potentially fatal bleeding from the ventriculotomy site. The transmural mattress sutures incorporating ventriculotomy patches required minimal bites toward the posteromedial papillary muscle and mitral annulus, thereby preserving the mitral valve function.

**Conclusions:**

Thus, the technique enhances the advantage of the left ventriculotomy in the repair of posterior septal rupture and avoids ventriculotomy-related morbidity.

## Background

The rupture of the posterior ventricular septum after acute inferior myocardial infarction is more challenging to repair than ruptures in other sites since it is less accessible and anatomically restricted.

**Fig. 1:**
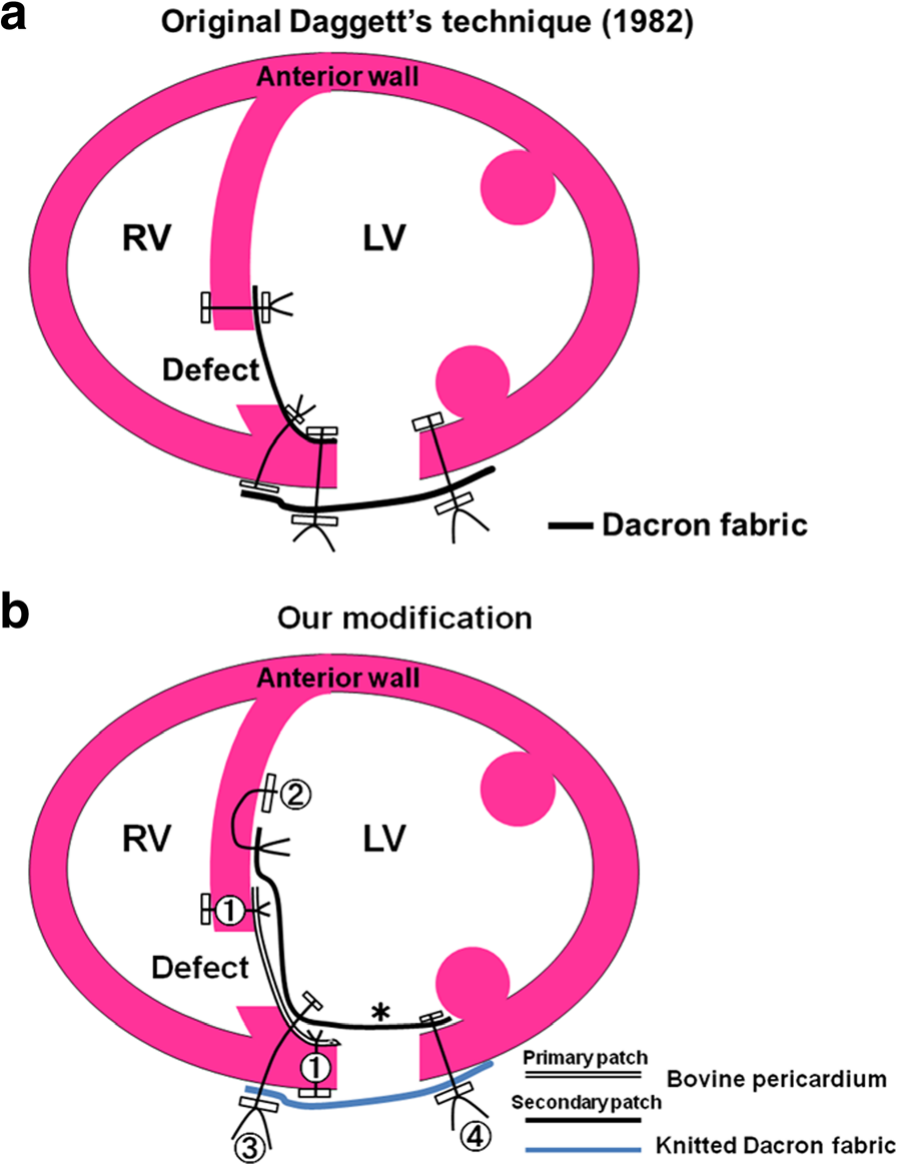
Schematic diagrams of the repair techniques. **a** The Daggett’s technique. The left ventricular defects are closed separately in a single layer. **b** The modification described in the text. Sutures are numbered and placed in numerical order. A cross section shows the secondary endoventricular patch (asterisk) closes both the septal and ventriculotomy defects separately with the hinge stitches between (③)

We modified Dr. Daggett’s trans-left-ventricular approach [1] (Fig. 1a) by introducing a large endoventricular patch which takes a major part in double-layered patch closure of both the septal defect and the left ventriculotomy defect (Fig. 1b). The technique enhances the advantage of the left ventriculotomy and avoids ventriculotomy-related morbidity.

## Case presentation

**Fig. 2:**
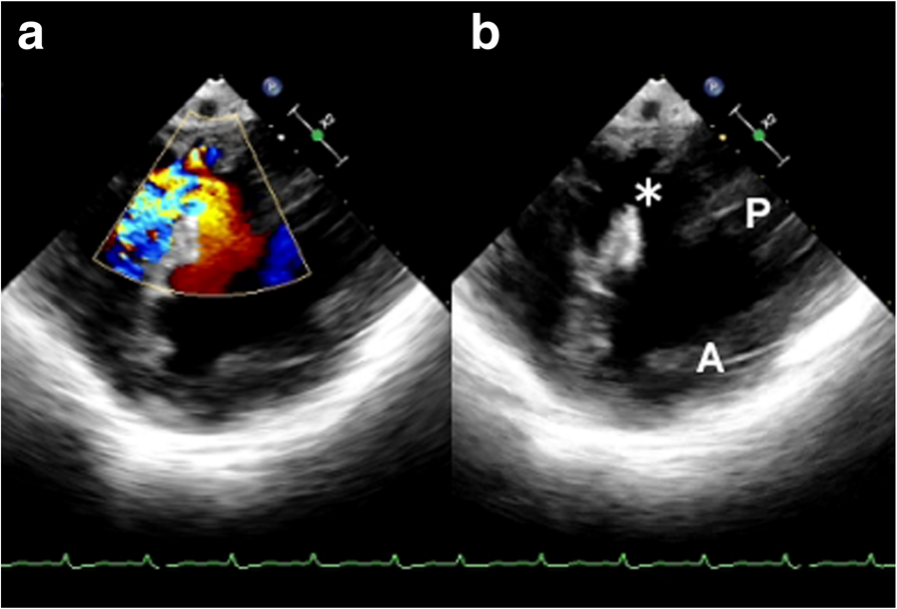
The preoperative transesophageal echocardiogram. **a** It showed transseptal interventricular communication with a shunting ratio of 2.4. **b** The defect (asterisk) located in the posterior septum close to the base of the posteromedial papillary muscle (P). A: anterior papillary muscle

A 67-year-old man presented with New York Heart Association class IV heart failure at the 10th day after he developed severe chest pain which receded in the next 7 days. On admission, the serum level of the MB fraction of creatinine kinase was 31 IU/L and the electrocardiogram showed Q waves in the inferior leads, suggesting a recent onset of inferior myocardial infarction. Coronary angiography showed an occluded thin distal right coronary artery with very slow retrograde filling from the main circumflex branch of the intact left coronary artery. The transesophageal echocardiogram showed a defect in the inferoposterior part of the ventricular septum and interventricular communication with a shunting ratio of 2.4 (Fig. 2). The left ventricular ejection fraction was 0.45. Catheter intervention was not indicated, but urgent surgery was required.

## Surgical technique

The heart was exposed through a median sternotomy and cardiopulmonary bypass established with bicaval cannulation and ascending aortic return.

The apex of the beating heart was then retracted cephalad with the aid of a couple of traction sutures placed in the bottom of the pericardial sac. The boundary between the septum and free wall was easily identified by gentle palpation of the diaphragmatic aspect of the empty heart. On total bypass with cold anterograde crystalloid cardioplegia, a left ventriculotomy was made parallel to the posterior descending coronary artery but closely to the boundary.

With balanced traction on pledgeted stay sutures placed in bilateral ventriculotomy edges, the posteromedial papillary muscle was fully visualized on the lateral edge of the ventriculotomy. Its base was not infarcted, but only 10 mm away from the ventriculotomy edge which was involved in infarction. The confronting anatomy should endanger direct closure of the ventriculotomy, suggesting the use of a patch for it. The ventricular septal defect and the area of necrotic muscle surrounding it were then exposed. Debridement of necrotic muscle was accomplished in order to define clearly the edges of the ventricular septal defect but with particular care avoiding overzealous removal of the muscle.

Interrupted mattress stitches of 4/0 polypropylene Teflon-pledgeted suture (① in Fig. 1b) were placed through the ventricular septum from the right ventricular side into the left ventricle. These stitches were continued until the free wall of the ventricle was reached, then the direction of the needle was switched to the outside-in fashion on the left ventricular free wall close to the right-side edge of the ventriculotomy. A 30 × 40 mm oval bovine pericardial sheet functioning as a primary closer of the septal defect was approximated to the ventricular septum and secured.

**Fig. 3:**
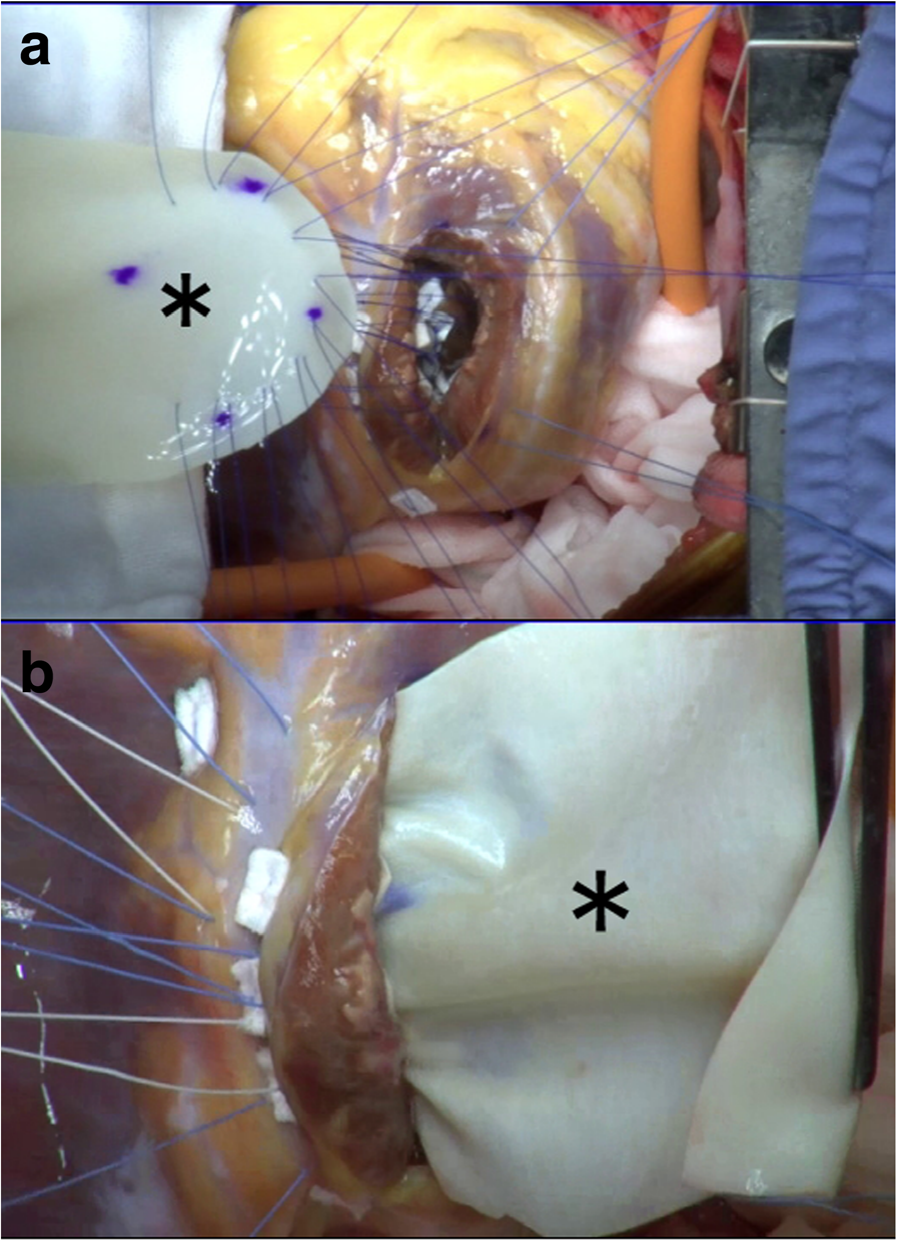
Steps of secondary closure of the septal defect. **a** A primary patch is undergoing additional covering of the comet-shaped secondary patch (asterisk). **b** The endoventricular polypropylene sutures (② in Fig. 1b) were succeeded by 2/0 braided sutures brought out to the interventricular groove (③ in Fig. 1b). This row was not only to complete the secondary closure of the septal defect but also to hold the external patch. It forms a hinge line between two parts of the secondary patch. The back side of the secondary patch (asterisk) is now shown

Interrupted mattress stitches of the same suture were then placed in the non-infarct area of the surrounding ventricular septum (② in Fig. 1b), 1 to 2 cm away from the edge of the septal defect already covered with the primary patch. These stitches were given with deep bites and then continued again until the free wall of the ventricle was reached. Here, a large patch of the “comet” shape was fashioned out of the rest of the bovine pericardial sheet. Those stitches were passed through the round edge (Fig. 3a), corresponding to the head of the “comet”, of this patch and secured it on the ventricular septum over the already patched septal defect.

Interrupted sutures of 2/0 braided synthetics with Teflon pledgets (③ in Fig. 1b) were then placed in a horizontal mattress fashion through the folded edge of the patch and then from the inside of the left ventricle to the outside on the interventricular groove. In the middle of this row, the stitches incorporated the primary patch into bitten layers. This row of mattress sutures in combination with the prior 4/0 polypropylene interrupted endocardial stitches (② in Fig. 1b) was to complete the secondary closure of the ventricular septal defect. But it was not only going to secure the septal portion of the patch but also going to hold the external patch for the following closure of the ventriculotomy defect. The remaining redundant portion of the secondary patch was then folded over laterally and fashioned for the closure of the ventriculotomy defect (Fig. 3b).

**Fig. 4:**
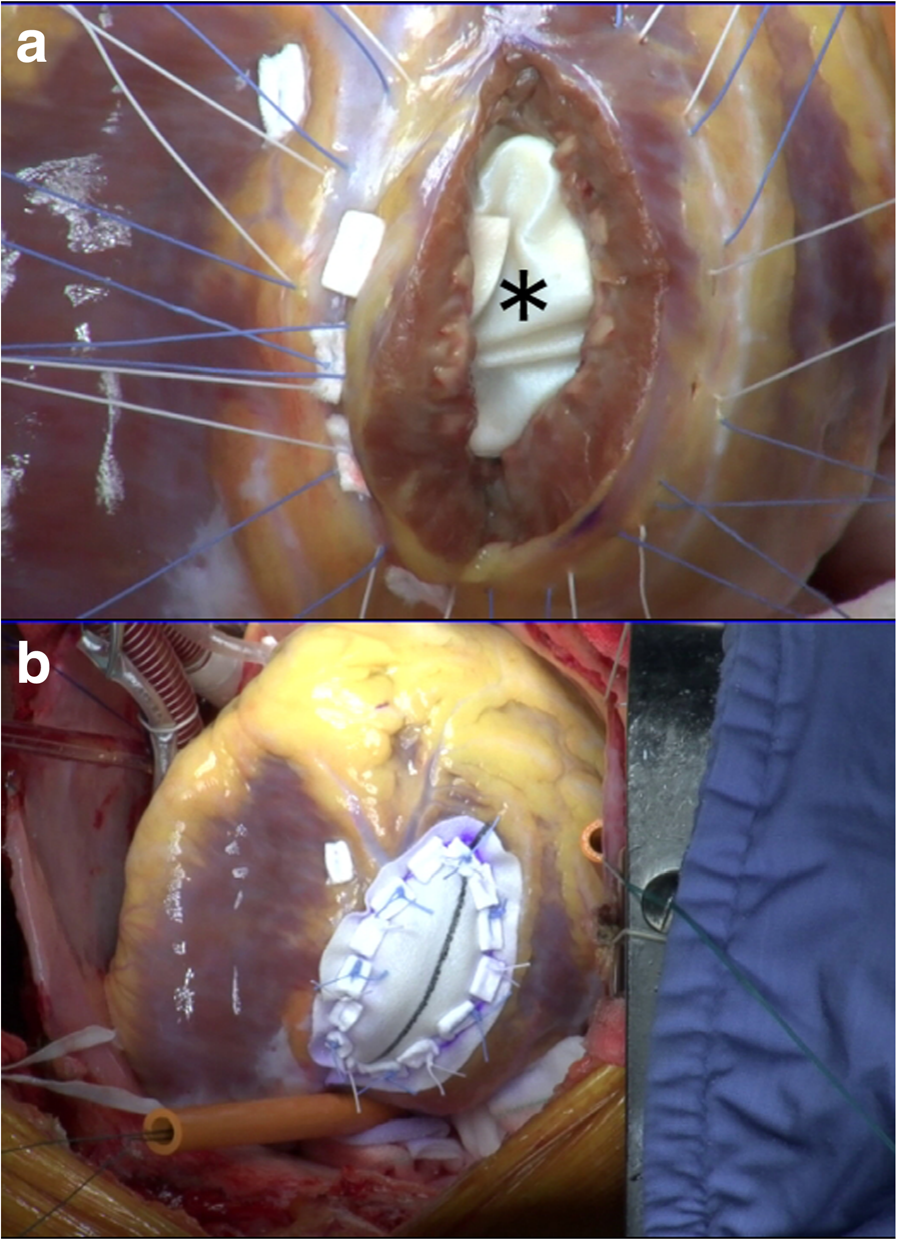
Closure of the free wall defect. **a** The “tail” of the secondary patch (asterisk) was folded over in the left ventricle and stitched from the inside to the outside with 2/0 braided sutures (④ in Fig. 1b). **b** An external patch of Dacron fabric with a width of 30 mm snugged down to close the ventriculotomy defect. The width of the patch should be slightly smaller than that of the stitched area to reduce tension of the internal patch

Another set of Teflon-pledgeted interrupted sutures of 2/0 braided synthetics (④ in Fig. 1b) were passed through the tail of this secondary patch and then from the inside of the left ventricle to the outside over the left side edge of the ventriculotomy. The base of the posteromedial papillary muscle was well off those stitches. When all the sutures were in place, the free wall defect created by the ventriculotomy was ringed about with 2/0 braided sutures and bottomed in 15 mm width by the secondary and now “common” endoventricular patch (Fig. 4a).

An appropriately but slightly undersized patch of collagen-sealed, velour knitted Dacron was fashioned to cover the ventricular free wall defect. The whole set of sutures secured the prosthesis to the heart with the buttress of Teflon pledgets (Fig. 4b).

The patient was weaned from cardiopulmonary bypass easily with moderate inotropic support and the intraaortic balloon pump. There was no additional stitches required for the ventriculotomy site. The operation time, pump time, and aortic cross-clamp time were 303, 200, and 151 min respectively. The bleeding amount was 200 mL whereas the 560 mL of concentrated red cells were transfused.

The patient made an uneventful recovery and was discharged on day 24. A postoperative echocardiogram at hospital discharge showed an ejection fraction of 0.43, trivial regurgitation of the mitral valve, and no residual interventricular shunting. He was kept on the anticoagulant (warfarin potassium). At follow-up 10 months later, he is in New York Heart Association class I with no sign of recurrence.

## Discussion

The trend toward multi-layered patch closure of the ischemic ventricular septal defect is now prevailing for the potential of decreasing recurrence [2, 3]. Our current practice then includes a primary septal closure using a local patch on the left ventricular side and a secondary closure with a large patch covering fully over the primary patch, no matter where and how large the defect is. The described technique certainly increases the complexity and procedural time by introducing an additional patch and interrupted sutures as compared to the original Daggett’s technique (Fig. 1). However, it enhances the great advantage of his technique which eliminates not only the everting sutures but also the tension on the friable muscle. We recognize that our modification never increases the scope of the indication for the original technique but surely augments its security. The primary closure could be more secure if the septal defect was “sandwiched” between two patches as was successfully employed in the right ventricular approach [4].

Monopatch repair by infarct exclusion is another option [5]. It is simple and applicable to a wide variety of septal ruptures. An akinetic free patch remains in the left ventricle but the geometric shape of the residual chamber should be much closer to normal than our technique where an endoventricular patch no smaller than the cross-sectional infarcted area remained. We therefore have reservations about the use of our technique for the patients with global impairment of the left ventricular function which should benefit from aggressive reduction of left ventricular volume.

The early repair often poses technical difficulty in creating secure closure of not only the septal defect but also the left ventricular incision especially when the adjacent left ventricular wall was extensively involved in infarction. Nevertheless, the left ventriculotomy approach is widely adopted since it provides optimal exposure of the septal defect.

The posterior wall of the left ventricle is invariably infracted in patients with posterior septal rupture; therefore, it is often unavoidable to incise the fully infarcted part. Direct suture closure of the infarcted left ventricular free wall always carries a substantial risk of suture line disruption and therefore requires large stitch bites which inevitably endanger adjacent structures. The ventriculotomy incision may be closed primarily only when the edges consist of either fully viable myocardium or reasonably mature scar tissue. In this context, a routine employment of the patch closure should be a safe option to eliminate the risk in this particular anatomical setting.

In our technique, the transmural mattress sutures incorporating both ventriculotomy patches require minimal bites toward the mitral valve annulus and the posteromedial papillary muscle, thereby preserving the mitral valve function. The patches on both the endocardial and the epicardial sides well avoid potentially fatal bleeding from the ventriculotomy site. At the moment, we prefer the Dacron patch for the external cover to the pericardial one by reason of baroresistance and durability.

In previous publications using a patch for the left ventriculotomy, the septal defect and the ventriculotomy defect were repaired separately with their own patches and often in layers [1, 6, 7]. Our technique expedites the process of closing two defects in two layers by employing a single continuous endoventricular patch with a set of transmural mattress sutures (③ and ④ in Fig. 1b) which are once tied down; the double-layered patch closure of both the ventricular septal defect and the ventriculotomy defect are fully and satisfactorily accomplished. The complexity increased by introducing the second patch layer to the original Daggett’s technique is minimized by using the identical patch for individual closure of two separate defects which otherwise should require two separate patches.

Diligent search of literatures failed to detect such a use of a single endoventricular patch in multilayered closure of both the septal defect and the left ventriculotomy defect. The placement of a continuous patch widely over the septum and left ventricular free wall might minimize geometric remodeling of the covered area.

## Conclusions

A continuous endoventricular patch covering both the ventricular septal defect and the ventriculotomy defect secured multi-patch repair of ischemic posterior ventricular septal rupture. Both defects were then individually closed in double layers, holding a single patch in common. The technique enhances the advantage of the left ventriculotomy and avoids ventriculotomy-related morbidity.
